# Pseudopheochromocytoma: An uncommon cause of malignant hypertension

**DOI:** 10.4103/0971-4065.57111

**Published:** 2009-07

**Authors:** S. M. Seck, E. F. Ka, A. Niang, B. Diouf

**Affiliations:** Nephrology Department of Teaching Hospital, Aristide Le Dantec, Dakar, Senegal

**Keywords:** Malignant hypertension, pseudopheochromocytoma, African

## Abstract

A 42-year-old black African patient was admitted in the emergency department with severe headache, dizziness, and visual problems. He had been treated for hypertension diagnosed eight months ago after a similar episode. He was taking atenolol 100 mg /day, amlodipine 10 mg/day, and a combination of lisinopril 20 mg/hydrochlorothiazide 12.5 mg daily but experienced several hypertension peaks and hypotension. He adhered to treatment and was neither using traditional herbal medication nor illicit drugs. He did not smoke, but used to drink 1–2 glasses of wine after dinner. At admission, his blood pressure was 235/145 mm of Hg. His body mass index was 25.5 kg/m^2^ and the waist/hip ratio was 0.9. Physical examination was unremarkable. Fundoscopic examination revealed hypertensive retinopathy. Biochemical and imaging explorations were compatible with diagnosis of pseudopheochromocytoma. Evolution was favourable after treatment with alpha-1 and beta-blokers.

## Introduction

Pseudopheochromocytoma is a rare cause of malignant hypertension that has been described since a long time in Caucasian patients.[1] However it is exceptionally reported in Africans.[2] We report the first case in a black Senegalese patient.

## Case Report

A 42-year-old black African was admitted in emergency department with severe headache, dizziness and visual problems. His medical history revealed existence of high blood pressure (HBP) discovered eight months earlier by his cardiologist after a similar episode after he came back from a business travel. He had been treated with many drugs (atenolol 100 mg/day, amlodipine 10 mg/day and a combination of lisinopril 20 mg/hydrochlorothiazide 12.5 mg daily). Despite this treatment, he experienced several hypertension peaks and hypotension during the last fours weeks. He adhered well to treatment and was neither on traditional herbal medication nor on illicit drugs. He did not smoke but used to drink 1-2 glasses of wine after dinner. Prior to this he was healthy and played basketball regularly at least three times a week.

Questioning revealed that he was married and lived happy with his wife and two children. He had been working 12 h per day as the financial analyst for an international group in stock exchange for the past ten years. He confessed having many additional hours last weeks in the context of international financial crisis but denied any particular fear, anxiety or emotional distress. Psychological evaluation was unremarkable.

At admission, his blood pressure was 235/145 mm Hg with 70 bpm of pulse, 98 kg of weight and 1.95 m of height. His body mass index was 25.5 kg/m^2^ and the waist /hip ratio was 0.9. Physical examination of systems was unremarkable. Fundoscopic examination of the eye at emergency room revealed hypertensive retinopathy stage 2 of Kirkendall classification. Electrocardiography and echocardiography found slight left ventricular hypertrophy with normal cardiac output. First line laboratory investigations are reported in Table 1.

**Table 1:** Laboratory investigations at emergency department

Results	Value
BUN	50 mg/dL
Serum creatinine	1.5 mg/dL
MDRDeGFR	66.0 ml/min
Serum albumin	38 g/l
Glucose	90 mg/dl
Na^+^	136 mmol/l
K^+^	3.8 mmol/l
Cl^−^	97 mmol/l
Ca^++^	2.05 mmol/l
CO_2_	25 mmol/l
Urinalysis: pH	6.6

BUN: blood urea nitrogen; RBC: red blood cells; WBC: white blood cells;eGFR: estimated glomerular filtration rate; MDRD: modification of diet in renal disease

At emergency department, he received nicardipine IV 2-4 mg/h and reinforced oral treatment with atenolol 150 mg/day, lisinopril 20 mg/day and hydrochlorothiazide 12.5 mg/day. Two days later, he was switched to oral nicardipine 150 mg/day while his systolic BP decreased below 180 mm of Hg. Then, he was transferred to nephrology department for further investigations.

Ultrasound and CT-scan showed kidneys with normal size, good cortical index, and normal vessels.

Table 2 shows further laboratory tests performed to look for a secondary cause of hypertension. These tests were repeated one week later but results were unchanged.

**Table 2:** Biochemical tests for secondary hypertension screening

	Tests values	Reference values
Plasma tests		
Thyroid stimulating hormone	2.47 mIU/l	0.45-4.9 mIU/l
Cortisol	430 nmol/l	245-845 nmol/l
Plasma renin	1.8 ng/ml	0.25-1.9 ng/ml
Plasma aldosterone	29 ng/100 ml	5-32 ng/100 ml
Plasma renin:aldosterone ratio	0.06	0.0625
Urine tests		
Aldosterone	20.5 mcg/l	5-24 mcg/l
Free cortisol	12 mcg/l	0-49 mcg/l
5-HIAA	3 mg/dl	0-8 mg/l
Normetanephrines	512 mcg/l	45-630 mcg/l
Metanephrines	220 mcg/l	35-400 mcg/l
Vanillyl mandelic acid	4 mg/l	0-7 mg/l

Diagnosis of pseudopheochromocytoma was suspected after results of these explorations

We stopped calcium channel blockers, continued the angiotensin-conversion enzyme inhibitor/diuretic and added doxazosin 1 mg daily and paroxetin 10 mg to patient's treatment. The patient was seen two weeks later for follow-up with his notebook where he recorded daily self-measured blood pressure at home with a semi-automatic sphygmomanometer. All blood pressure measures were under 140/90 mm of Hg like the one measured during consultation. A new fundoscopic eye examination showed regression of retinopathy to stage 1 and his renal function normalized. He had the same overcrowded working hours but felt better without any symptom. Two months later, we reduced atenolol to 50 mg/day to prevent hypotension episodes and he had a stable blood pressure within normal ranges even after exercise.

## Discussion

In the presence of malignant hypertension in this young patient, we looked for secondary causes of hypertension. We first ruled out renal diseases (glomerular and vascular) that are common aetiologies of secondary hypertension whether in black and non-black patients.[3,4] Renal impairment in our patient was related to malignant hypertension that is frequently associated with renal vascular damage due to markedly activated renin angiotensin system.[5] This was confirmed by the absence of glomerular injury signs (albuminuria, hematuria, hypoalbuminemia) on urinalysis and normal morphology of kidneys and vessels with CT-scan examination.

Other causes of secondary hypertension such as primary hyperaldosteronism, hyperthyroidism, hypercorticism, and endocrine neoplasms were excluded by hormonal explorations. Pheochromocytoma is as a very rare etiology of hypertension[6] but represents the second cause of curable hypertension in our context.[4] In spite of suggestive clinical symptoms in this patient, cathecholamine-secreting pheochromocytoma was excluded by normal results of plasma and urine tests. Other causes of liable elevation in plasma or urine cathecholamines such as obstructive sleep apnea,[7] pre-eclampsia,[8] anti-Parkinsonian drugs[9] or cocaine use had also been discussed.

Panic attack, alcohol withdrawal and carcinoid syndromes are other situations that can explain paroxysmal hypertension in the absence of overt catecholamine excess.[10] None of these conditions were found in our patient who was previously healthy without any medication and denied any alcohol or illicit drug abuse. Finally, presence of paroxysmal hypertension without biochemical evidence of catecholamine excess in our patient met criteria for a diagnosis of pseudopheochromocytoma.

Pseudopheochromocytoma was first described as an apparently essential paroxysmal hypertension without biochemical evidence of catecholamine excess.[1] Many cases of such hypertension have been reported since 1981, but this is the first one in a black African.[2] This ethnic group is known to have higher prevalence and worse outcomes of malignant hypertension than Caucasians.[11] Early descriptions emphasized the emotional underlying background that often triggers hypertension peaks.[12,13] Anxiety and psychological instability may play a key role in onset of severe hypertension in patients at risk.[12,14] Even if our patient denied any uncommon anxiety or distress, one can suspect a link between hypertension and his emotional state. His first episode of paroxysmal hypertension eight months before diagnosis occurred after a crucial business travel and the last one was concomitant with more stress at job because of unpredictable international financial situation.

Retrospectively, response to therapy confirmed diagnosis. We rapidly observed a good response to combination of alpha-1 and beta-blockers with psychological support and antidepressant drug therapy. This multidisciplinary approach is recommended for the management of pseudo-pheochromocytoma.[14]

## Conclusion

Pseudopheochromocytoma is a rare cause of high blood pressure. It should be discussed in any case of paroxysmal hypertension without evidence of catecholamine excess, but after ruling out other classical differential diagnosis. A particular underlying psychological context can help but overt anxiety is sometimes absent. Treatment relies on alpha-1- and beta-blockers combined with psychopharmacological interventions.
